# Added value of dual-phase ^99m^Tc-HYNIC-FAPI-04 SPECT/CT in postoperative follow-up of colorectal cancer

**DOI:** 10.3389/fonc.2025.1500273

**Published:** 2025-06-16

**Authors:** Donghua Sun, Yan Liu, Renhua Hou, Jianan Li, Ye Peng, Yingqiu Wang

**Affiliations:** ^1^ Department of Nuclear Medicine, Yangpu Hospital, School of Medicine, Tongji University, Shanghai, China; ^2^ Department of Nuclear Medicine, the First Affiliated Hospital of Naval Medical University, Shanghai, China

**Keywords:** 99mTc -HYNIC-FAPI-04, SPECT/CT, dual-phase imaging, colorectal cancer, postoperative monitoring

## Abstract

**Objective:**

To assess the added value of dual-phase ^99m^Tc-HYNIC-FAPI-04 single-photon emission computed tomography/computed tomography (SPECT/CT) compared with single-phase imaging in postoperative follow-up of colorectal cancer (CRC).

**Methods:**

Early and delayed ^99m^Tc-HYNIC-FAPI-04 SPECT/CT imaging were undertaken in 21 patients with CRC after surgery. The mean radioactivity count of the lesion site and abdominal aorta in secondary imaging was delineated and measured. The early and delayed target background ratio (TBR) and retention index (RI) were calculated, respectively. The results of biopsy or postoperative pathology, clinical and imaging follow-up were used as the “gold standard” of tumor metastasis. Lesions were divided into “benign” and “malignant” groups. The uptake rate and metabolic rate of ^99m^Tc-HYNIC-FAPI-04 in the two groups were observed. The diagnostic efficiency of early imaging, delayed imaging, and their combination for prediction of benign and malignant lesions was compared using receiver operating characteristic (ROC) curves.

**Results:**

Fifty-five lesions with abnormal uptake of ^99m^Tc-HYNIC-FAPI-04 were found by SPECT/CT. Compared with TBR-early, TBR-delayed in malignant group was increased (4.07 ± 1.65 *vs*. 4.36 ± 1.86, *P* = 0.040), whereas TBR-delayed in the benign group was decreased significantly (2.96 ± 0.85 *vs*. 2.66 ± 0.77, *P* < 0.001). The mean count of radioactivity in the delayed phase, TBR-early, TBR-delayed, and RI in the malignant group were higher than those in the benign group (*P =* 0.005, <0.001, and <0.001, respectively). ROC curves showed that combined application of TBR-early, TBR-delayed, and RI had an AUC of 0.889, specificity of 0.900, and sensitivity of 0.844, and its diagnostic efficiency was superior to that of TBR-delayed (*P =* 0.026) and TBR-early (*P <*0.001).

**Conclusions:**

Dual-phase ^99m^Tc-HYNIC-FAPI-04 SPECT/CT could be used for the detection of metastatic lesions after CRC surgery. It has a higher auxiliary role than single-phase imaging for the differential diagnosis of benign and malignant lesions after CRC surgery.

## 1 Introduction

Colorectal cancer (CRC) is a common tumor of the human digestive system. CRC has the third highest incidence and is the second most-common cause of death among malignant tumors (1). Hence, CRC poses a serious threat to human health.

Radical surgery is first-line treatment for CRC. However, recurrence or metastasis of the tumor is observed in about 30–50% of patients postoperatively, most of which occurs within 3 years after surgery (2). Postoperative monitoring is very important in the survival of patients suffering from CRC.

In the past decade, positron emission tomography/computed tomography (PET/CT) has gradually become an important method of postoperative monitoring of CRC due to its high sensitivity and specificity in cancer diagnosis (3–5). With application of the novel molecular probes such as fibroblast activation protein inhibitors (FAPIs), the diagnostic advantage of PET/CT in CRC has become pronounced (6–8). Fibroblast activation protein (FAP) is overexpressed in cancer-associated fibroblasts, which are the predominant components in the stroma of epithelial neoplasms such as CRC. Radionuclide-labeled FAPI PET/CT has shown high uptake of FAP at tumor sites, whereas background values in muscle and blood pools are low, showing very high uptake and image contrast. Therefore, radionuclide-labeled FAPI PET/CT has become a new method for cancer diagnosis (9, 10). However, the expense of follow-up using PET/CT can cause a high economic burden to patients. More accessible methods are needed in the postoperative monitoring of CRC.

The principle of radionuclide-labeled FAPI of SPECT/CT is similar to that of PET/CT. SPECT/CT has a slightly lower diagnostic efficiency than PET/CT for malignant lesions, but its cost is low and the equipment is more popular, which is conducive for clinical promotion and application. Preliminary studies have shown that, in technetium-99m (^99m^Tc)-labeled FAPI SPECT/CT, the target background ratio (TBR) is high in tumors with high expression of FAP (11, 12). Those data suggest the diagnostic potential of ^99m^Tc-labeled FAPI SPECT/CT in CRC.

We wished to improve the diagnostic efficiency of ^99m^Tc-labeled FAPI SPECT/CT. Hence, we investigated the diagnostic value of dual-phase ^99m^Tc-HYNIC-FAPI-04 SPECT/CT in postoperative patients with CRC.

## 2 Materials and methods

### 2.1 Study design

We investigated the diagnostic value of dual-phase ^99m^Tc-HYNIC-FAPI-04 SPECT/CT in postoperative patients with CRC.

The protocol for this prospective study was approved (LL-2023-SCI-010) by the Ethics Committee of Yangpu Hospital Affiliated to Tongji University (Shanghai, China). Enrolled patients provided written informed consent before study initiation.

Postoperative patients with CRC were recruited consecutively from January 2023 to September 2023. All patients with suspected recurrence or metastases had not undergone radiotherapy, chemotherapy, or targeted therapy previously. Early (1 h) and delayed (3 h) imaging (^99m^Tc-HYNIC-FAPI-04 SPECT/CT) was undertaken. Whole-body imaging, as well as thoracic, abdominal, and pelvic SPECT/CT fusion imaging, were done at both phases. The malignancy of lesions was confirmed by tissue biopsy or repeated postoperative pathological, clinical, and imaging follow-up.

### 2.2 Imaging protocol for ^99m^Tc-HYNIC-FAPI-04

To prepare ^99m^Tc-HYNIC-FAPI-04, HYNIC-FAPI-04 (Shanghai Nice-labeling Biotechnology, Shanghai, China) was radiolabeled with ^99m^Tc-NaTcO_4_ (Shanghai Atom Kexing Pharmaceuticals, Shanghai, China) according to the instruction manual. The radiochemical purity was >95% for use.

The patient was supine and injected (i.v.) with ^99m^Tc-HYNIC-FAPI-04 740MBq (20 mCi). Then, the patient underwent early and delayed whole-body imaging and SPECT/CT fusion imaging at 1 h and 3 h, respectively, using the Discovery NM/CT 670 system (GE Healthcare, Chicago, IL, USA). For the collimator, the matrix was 256 × 256, magnification was 1.0, and bed speed was 15 cm/min. For SPECT, the imaging range was the chest, abdomen, and pelvic cavity at 32 frames, 15 s/frame, double-probe automatically rotated 180°, iterative Flash 3D reconstruction, with an iteration number of 8, and subset of 8. For simultaneous fusion CT, the voltage was 130 kV, current was 120 mA, and pitch was 1.0.

### 2.3 Image interpretation

Dual-phase ^99m^Tc-HYNIC-FAPI-04 images were analyzed by two physicians with 10 years of experience in nuclear medicine. Local radioactive accumulation in ^99m^Tc-HYNIC-FAPI-04 whole-body imaging and SPECT/CT fusion images with a space-occupying lesion on CT were identified as “positive”. To determine TBR in early phase and delayed phase, the largest axial-section of positive lesions was selected as the region of interest (ROI) as the target uptake, and the abdominal aorta at the level of the celiac trunk was selected as the background. TBR was calculated by comparing the average radioactive counts of target sites with the background, as shown in Equation 1.


(1) $$\text{TBR }(\text{early},\text{delayed})=\frac{\text{Average counts in the lesion}}{\text{Average counts in the aorta}}$$

The difference in TBR between early and delayed images of all lesions was determined by the retention index (RI), calculated as shown in Equation 2.


(2) $$\text{RI}=\frac{\text{TBR}-\text{delayed }-\text{TBR}-\text{early}}{\text{TBR}-\text{early}}$$

Lesions with abnormal uptake of ^99m^Tc-HYNIC-FAPI-04 were divided into “benign” (postoperative scar, pulmonary fibrosis) and “malignant” (metastasis) groups according to pathology, medical history, and imaging results.

### 2.4 Statistical analyses

Statistical analyses were undertaken using SPSS 26.0 (IBM, Armonk, NY, USA). Measurement data are expressed as the mean ± standard deviation (SD). Student *t*-tests were used to analyze the mean radioactivity count, TBR, and RI of the lesion site and background between the two groups in the early phase and delayed phase. Paired *t*-tests were employed to compare early TBR and delayed TBR within a group. Receiver operating characteristic (ROC) curves were used to analyze the diagnostic efficacy of TBR-early, TBR-delayed, RI, and their combined application in the diagnosis of malignant lesions after CRC. *P <*0.05 was considered significant.

## 3 Results

### 3.1 Baseline characteristics of patients with CRC receiving ^99m^Tc-HYNIC-FAPI-04 SPECT/CT

Twenty-one patients formed the study cohort (17 of them were male). The mean age was 59.71 ± 12.76 years. Primary tumors were in the ascending colon (4/21, 19.05%), transverse colon (5/21, 23.81%), sigmoid colon (7/21,33.33%), or rectum (5/21, 23.81%). The median postoperative course was 360.00 (range, 30–1800 days). All metastases were evaluated by biopsy or postoperative pathology, clinical and imaging follow-up. Dual-phase ^99m^Tc-HYNIC-FAPI-04 SPECT/CT was tolerable in all patients. The mean duration of follow-up since ^99m^Tc-HYNIC-FAPI-04 SPECT/CT was 105.38 ± 44.58 days (**Table 1**).

**Table 1:** Demographic and clinical characteristics of patients with CRC undergoing ^99m^Tc-HYNIC-FAPI-04 SPECT/CT.

Characteristic	No.
Sex	
Male, n (%)	17 (80.95%)
Female, n (%)	4 (19.05%)
Age (years), mean ± SD	59.71 ± 12.76
Site and pathology of primary tumor	
Ascending colonic adenocarcinoma, n	4 (19.05%)
Transverse colonic adenocarcinoma,	5 (23.81%)
Sigmoid colonic adenocarcinoma	7 (33.33%)
Rectum adenocarcinoma	5 (23.81%)
Postoperative course(days), mean ± SD	508.57 ± 540.62
Follow-up duration (days), mean ± SD	105.38 ± 44.58

SD, standard deviation.

**Figure 1:**
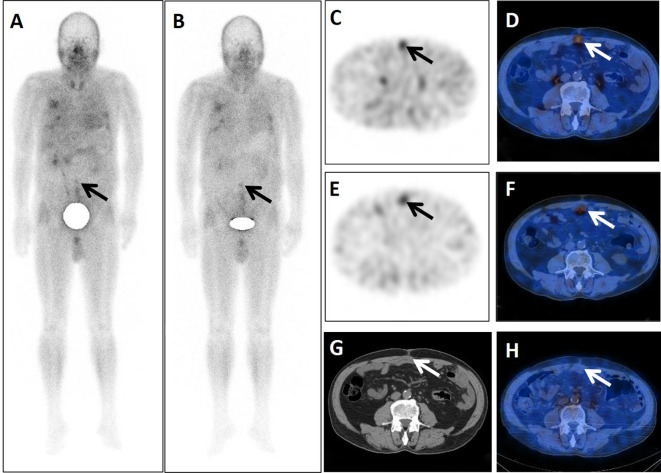
A 71-year-old postoperative male patient with a tumor in the sigmoid colon underwent dual-phase ^99m^Tc-FAPI-04 SPECT/CT. The early anterior whole-body MIP image **(A)** showed abnormal accumulation of radioactivity in the middle lower abdomen (arrow). The corresponding axial SPECT image (**C**, arrow) and fused ^99m^Tc-FAPI-04 SPECT/CT image (**D**, arrow) showed ^99m^Tc-FAPI-04 uptake in the lesion with TBR = 3.10. The lesions were dimmer in the delayed anterior whole-body maximal intensity projection (MIP) image (**B**, arrow), corresponding axial SPECT image (**E**, arrow), and fused ^99m^Tc-FAPI-04 SPECT/CT image (**F**, arrow) with TBR = 2.84. The axial CT image **(G)** showed an irregular postoperative scar measuring 48 mm × 21 mm (arrow). Six months later, this scar did not take up ^99m^Tc-FAPI-04 abnormally (**H**, arrow).

**Figure 2:**
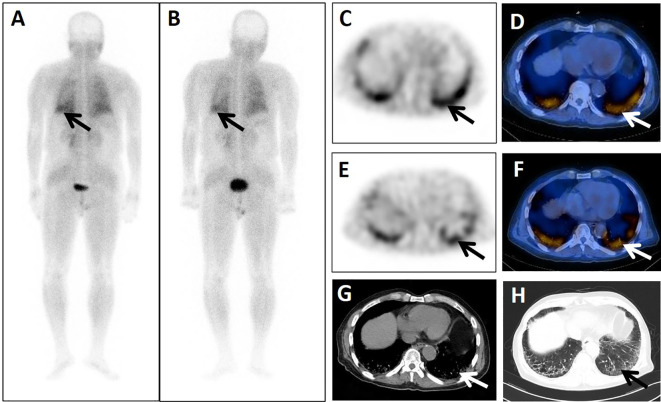
A 74-year-old male patient presented 4 years after surgery for rectal cancer with a history of pulmonary interstitial fibrosis of duration >2 years. He underwent dual-phase ^99m^Tc-FAPI-04 SPECT/CT. The early post whole-body MIP image **(A)** showed abnormal accumulation of radioactivity in the lower left lung (arrow). The corresponding axial SPECT image (**C**, arrow) and fused ^99m^Tc-FAPI-04 SPECT/CT image (**D**, arrow) of the lesions showed ^99m^Tc-FAPI-04 uptake with TBR = 3.60. The lesions were dimmer in the delayed post-whole-body MIP image (**B**, arrow), corresponding axial SPECT image (**E**, arrow), and fused ^99m^Tc-FAPI-04 SPECT/CT image (**F**, arrow) with TBR = 3.15. The axial CT image **(G)** showed abnormal metabolic lesions in the interstitial lesions of the bilateral lung base (arrow). Simultaneous high-resolution CT (**H**, arrow) confirmed the diagnosis of pulmonary interstitial fibrosis.

**Figure 3:**
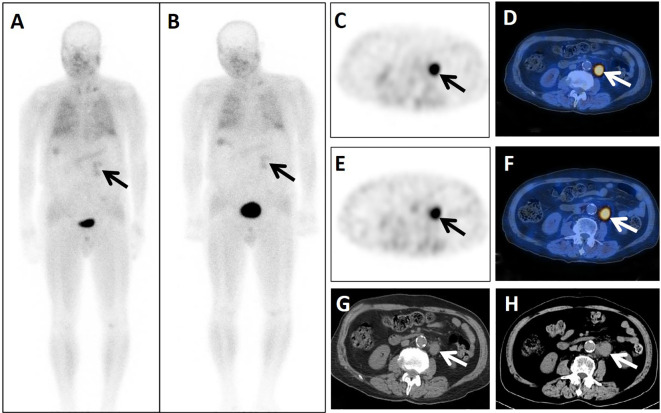
A 74-year-old male patient presented 4 years after surgery for rectal cancer. He underwent dual-phase ^99m^Tc-FAPI-04 SPECT/CT. The early anterior whole-body MIP image (**A**, arrow) showed abnormal accumulation of radioactivity in the left upper abdomen. The corresponding axial SPECT image (**C**, arrow) and fused ^99m^Tc-FAPI-04 SPECT/CT image (**D**, arrow) of the lesion showed high uptake of ^99m^Tc-FAPI-04 with TBR = 7.06. The lymph node was shown more clearly in the delayed anterior whole-body MIP image (**B**, arrow), corresponding axial SPECT image (**E**, arrow), and fused ^99m^Tc-FAPI-04 SPECT/CT image (**F**, arrow). The lesion showed higher uptake of ^99m^Tc-FAPI-04 with TBR = 7.68. The axial CT image (**G**, arrow) showed a lymph node measuring 32 mm × 22 mm in the left beside of abdominal aorta. Lymph-node metastasis was confirmed by follow-up CT (**H**, arrow) 4 months later, measuring 39 mm × 26 mm with increased levels of the tumor markers CEA (18.69 ng/mL), CA724 (27.77 IU/mL), and CA199 (48.89 U/mL).

**Figure 4:**
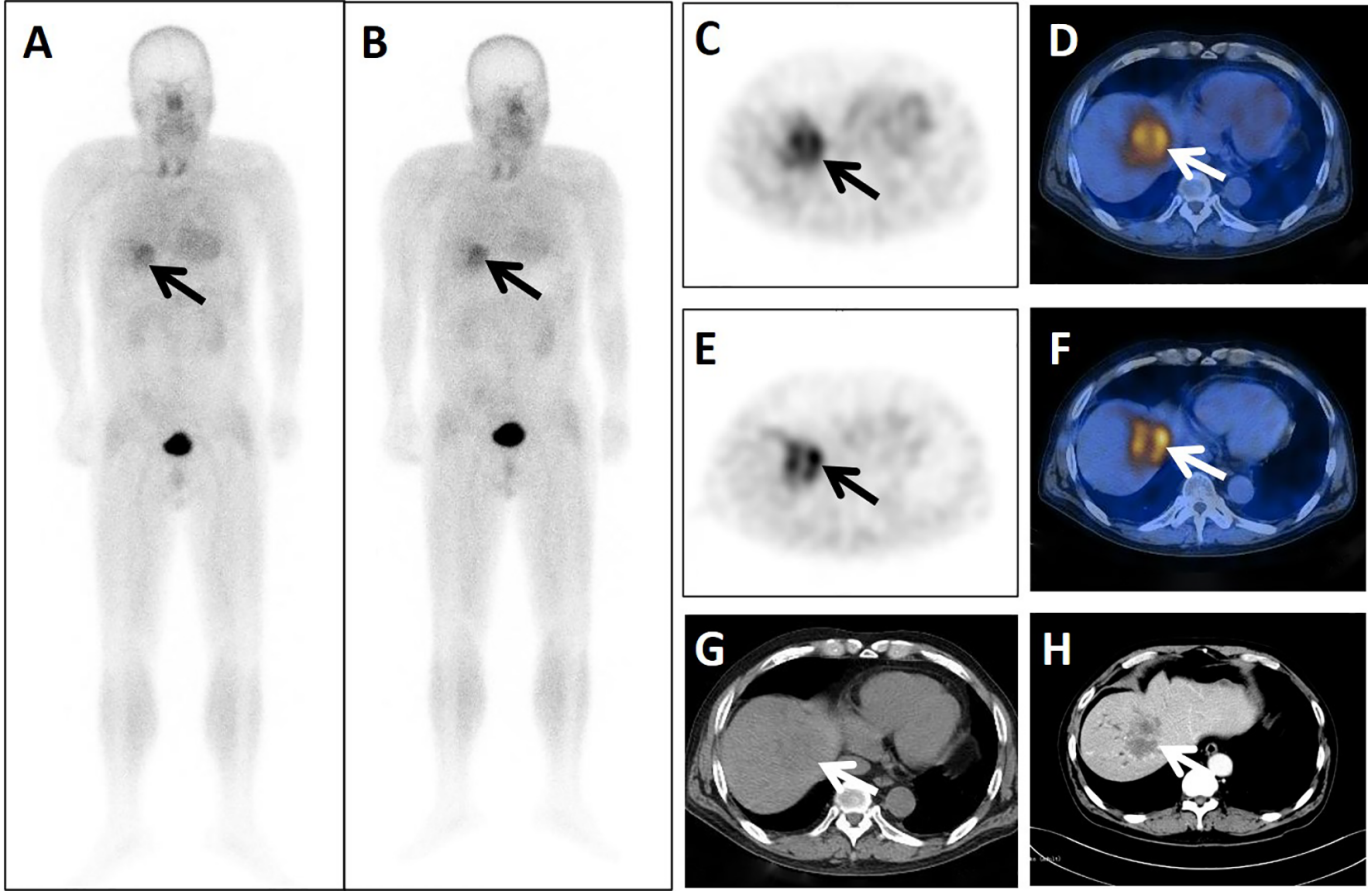
A 24-year-old postoperative male patient with cancer of the transverse colon cancer underwent dual-phase ^99m^Tc-FAPI-04 SPECT/CT. The early anterior whole-body MIP image (**A**, arrow) showed abnormal accumulation of radioactivity in the liver region. The corresponding axial SPECT image (**C**, arrow) and fused ^99m^Tc-FAPI-04 SPECT/CT image (**D**, arrow) of the lesion showed high uptake of ^99m^Tc-FAPI-04 with TBR = 6.38. The lesion was shown more clearly in the delayed anterior whole-body MIP image (**B**, arrow), corresponding axial SPECT image (**E**, arrow), and fused ^99m^Tc-FAPI-04 SPECT/CT image (**F**, arrow) with high uptake and TBR = 6.88. Axial CT image (**G**, arrow) showed a huge low-density mass measuring 67 mm × 53 mm. The lesion was confirmed to be liver metastasis by enhanced CT (**H**, arrow), with increased levels of tumor markers CA50 (182.95 ng/mL), CA242 (217.53 IU/mL), and CA199 (2035.75 U/mL).

Among the 21 patients, metastasis occurred in 13 cases, and no recurrence or metastasis occurred in 8 cases. Fifty-five sites had abnormal uptake of ^99m^Tc-HYNIC-FAPI-04 according to SPECT/CT fusion imaging: 10 benign lesions (**Figures 1**, **2**) and 45 malignant lesions (**Figures 3**, **4**) (**Table 2**).

**Table 2:** Positive lesion sites for ^99m^Tc-HYNIC-FAPI-04.

Location	Benign		Malignant					
	Scar	Pulmonary fibrosis	Liver	Lymph node	Lung	Spleen	Adrenal gland	Bone
n	6	4	18	18	6	1	1	1

### 3.2 ^99m^Tc-HYNIC-FAPI-04 has higher stability in postoperative metastatic lesions of CRC than in benign lesions

**Figure 5:**
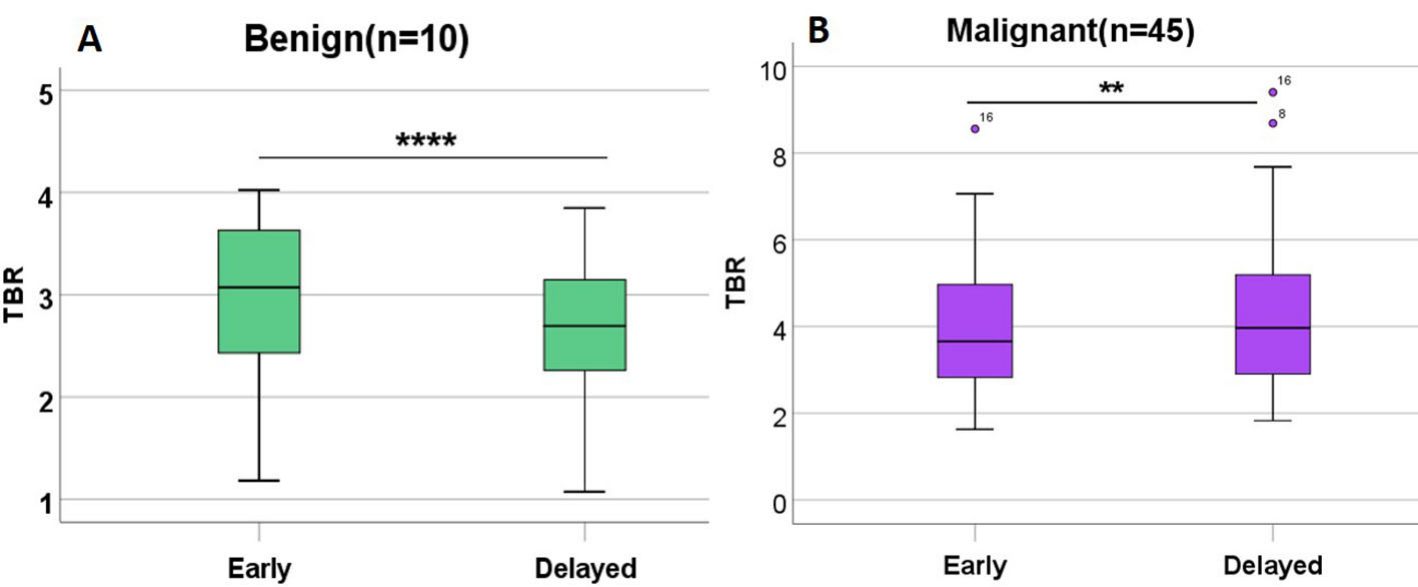
TBR in benign lesions and malignant lesions in patients with CRC undergoing ^99m^Tc-FAPI-04 SPECT/CT. TBR-delayed was lower than TBR-early in the benign group **(A)**. TBR-delayed was higher than TBR-early in the malignant group **(B)**. **P<0.05; ****P<0.001.

In the benign group, TBR-delayed (2.66 ± 0.77) was lower than TBR-early (2.96 ± 0.85, *P* < 0.001). In the malignant group, TBR-delayed (4.36 ± 1.86) was higher than TBR-early (4.07 ± 1.65, *P =* 0.040) (**Figure 5**). In the early phase, the mean radioactivity count in the benign group was 778.80 ± 318.82, and 1011.81 ± 457.70 in the malignant group, but this difference was not significant (*P =* 0.132). In the delayed phase, the mean radioactivity count in the malignant group was significantly higher than that in the benign group (760.00 ± 349.31 *vs*. 468.40 ± 195.55, *P* = 0.014). TBR-early (4.07 ± 1.65 *vs*. 2.96 ± 0.85, *P* = 0.005), TBR-delayed (4.36 ± 1.86 *vs*. 2.66 ± 0.77, *P* < 0.001), and RI (0.91 ± 0.25 *vs*. −0.10 ± 0.04, *P <* 0.001) in the malignant group were higher than those in the benign group (**Table 3**).

**Table 3:** Parameters of dual-phase ^99m^Tc-FAPI-04 SPECT/CT for the identification of benign and malignant lesions in patients with CRC.

Group	n	ELC	EBC	TBR-early	DLC	DBC	TBR-delayed	RI
Benign	10	778.80 ± 318.82	267.41 ± 75.86	2.96 ± 0.85	468.40 ± 195.55	175.74 ± 47.56	2.66 ± 0.77	-0.10 ± 0.04
Malignant	45	1011.81 ± 457.70	250.85 ± 53.14	4.07 ± 1.65	760.00 ± 349.31	175.78 ± 44.50	4.36 ± 1.86	0.91 ± 0.25
*P*		0.132	0.415	0.005	0.014	0.998	<0.001	<0.001

ELC, early lesion counts; EBC, early background counts; DLC, delayed lesion counts, DBC, delayed background counts.

### 3.3 Diagnostic efficiency of dual-phase ^99m^Tc-HYNIC-FAPI-04 SPECT/CT in benign and malignant lesions after CRC surgery

**Figure 6:**
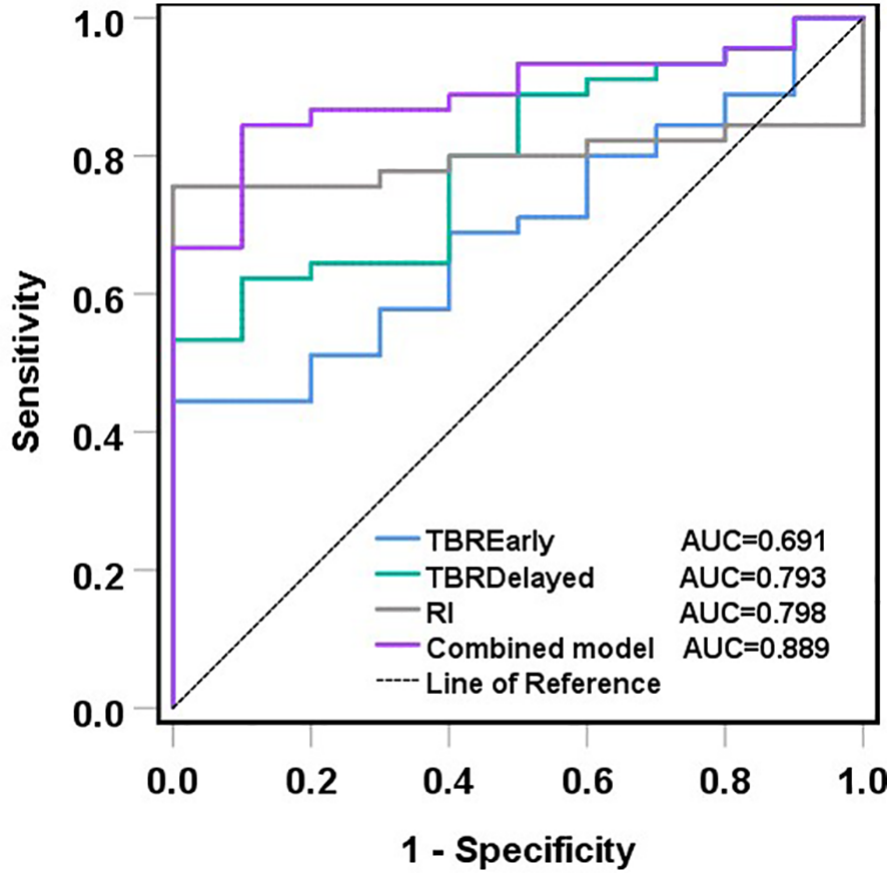
ROC curves of TBR-early, TBR-delayed, RI and their combination in the diagnosis of malignant lesions after CRC surgery. ROC analyses showed that the combined model had the biggest AUC, and its diagnostic efficiency was superior to that of TBR-delayed (P = 0.026) and TBR-early (P < 0.001).

ROC curves showed that TBR-delayed (*P* = 0.004) and RI (*P* = 0.003) had higher diagnostic efficacy for postoperative malignant lesions of CRC. The area under the ROC curve (AUC) of TBR-early, TBR-delayed, and RI was 0.691, 0.793, and 0.798, respectively. Combined application of TBR-early, TBR-delayed, and RI had an AUC of 0.889, specificity of 0.900, and sensitivity of 0.844, and its diagnostic efficiency was superior to that of TBR-delayed (*P* = 0.026) and TBR-early (*P* < 0.001) (**Table 4**; **Figure 6**).

**Table 4:** Diagnostic efficacy of TBR-early, TBR-delayed, and RI and their combination for prediction of the postoperative malignant lesions of CRC.

Prediction model	AUC	95%CI	Specificity %	Sensitivity %	Cutoff	*P*
TBR-early	0.691	0.540-0.842	100	44.4	4.027	0.061
TBR-delayed	0.793	0.664-0.922	100	53.3	3.876	0.004
RI	0.798	0.685-0.911	100	75.6	-0.031	0.003
Combined model	0.889	0.800-0.978	90	84.4	0.7833	<0.001

## 4 Discussion

CRC is a serious threat to human health. CRC appears to be developing at younger ages (13). In postoperative patients with CRC, periodic follow-up and utilization of examinations to detect tumor recurrence and metastasis are essential for their survival. Several methods are used commonly for postoperative monitoring of CRC: serum markers of tumors, epigenetic assays (14, 15), endoscopy, and radiology. However, none of these methods can reflect the condition accurately and completely due to their limitations.

Imaging studies are an important part of evaluating the screening, staging, and monitoring of patients with CRC. CT and magnetic resonance imaging (MRI) are the most widely used methods in routine imaging. In recent years, with the application of new molecular probes, nuclear-medicine examination has gradually become an important means for the diagnosis and staging of tumors (16, 17). SPECT/CT is the most popular method of nuclear-medicine examination. With the emergence of various new types of tracer, SPECT/CT can be done rapidly with multi-temporal and multi-frequency features. SPECT/CT can become a good method for the qualitative diagnosis, staging, as well as monitoring the recurrence and metastasis of tumors (18–20).

Various FAPI imaging agents labeled by a single-photon radionuclide, ^99m^Tc, have been used in tumor imaging (11, 12). In the present study, dual-phase ^99m^Tc-HYNIC-FAPI-04 was carried out in 21 patients after CRC surgery. Fifty-five sites with abnormal uptake of ^99m^Tc-HYNIC-FAPI-04 were found, 45 of which were malignant lesions and 10 of which were benign lesions. TBR-delayed was lower than that of TBR-early in the benign group, whereas TBR-delayed was higher than that of TBR-early in the malignant group. Meanwhile, we compared the parameters between these two groups of lesions. In the early phase, no significant difference was found in the radioactivity count at the lesion site. In the delayed phase, the radioactivity count was significantly higher in the malignant group than that in the benign group. TBR-early, TBR-delayed, and RI were significantly higher in the malignant group than those in the benign group. These results suggested a higher uptake and lower metabolic rate of ^99m^Tc-HYNIC-FAPI-04 in malignant lesions than those in benign lesions, data which are consistent with results from previous studies (21, 22).

^99m^Tc-labeled FAPI SPECT/CT also has a high TBR, which is suitable for the diagnosis of tumors with high FAP expression. The new molecular probe FAPI has been shown to be useful for imaging tumors with high expression of FAP by radionuclide labeling through different targets and chelators. However, studies have shown that FAP has physiological uptake in multiple organs, intestinal and urinary metabolism, and has significant expression in several benign diseases (23–25). These nonspecific expressions of FAP would affect the identification of benign and malignant diseases especially in single-phase imaging. Therefore, dual-phase ^99m^Tc-HYNIC-FAPI-04 SPECT/CT was used in the present study, and ROC curves were used to compare and analyze the diagnostic efficacy for patients with benign and malignant lesions after CRC surgery. We found that use of TBR-early alone for the diagnosis of the malignant lesions after CRC surgery was less effective and hampered distinguishing malignant lesions from benign lesions. TBR-delayed and RI could improve the diagnostic efficiency, and a combination of TBR-early, TBR-delayed, and RI improved the diagnostic efficiency. Similarly, Miyake et al. (26), found that dual-phase PET/CT was helpful to distinguish postoperative recurrence and the scarring wrought by CRC. Qiao et al. (27) found that uptake of ^18^F-AlF-NOTA-FAPI-04 in inflammatory lesions were significantly lower than that in malignant lesions. Application of dual-phase imaging may be valuable for distinguishing malignant and various inflammatory manifestations. In the present study, different uptake levels and metabolic rates of ^99m^Tc-HYNIC-FAPI-04 in benign and malignant lesions was observed by dual-phase imaging, so benign lesions could be differentiated from malignant lesions.

More than 1.9 million new cases of CRC (including the anus) and 935,000 deaths were estimated to occur in 2020 (28). CT and MRI have a poor ability to distinguish postoperative scarring from original recurrence of CRC, which is significantly lower than that observed using ^18^F-FDG PET/CT (29–31). PET/CT using ^18^F-FDG has a key role in this setting, having been included in the standard-of-care in the restaging of patients suffering from CRC (32). PET using ^18^F-FDG is also useful for detecting CRC in patients presenting with an unknown primary tumor (33). In recent years, with the application of different positron nuclide-labeled FAPI probes (e.g., ^68^Ga-FAPI-04), the diagnostic sensitivity of PET/CT for colorectal malignancies has improved (34, 35). In the present study, dual-phase ^99m^Tc-HYNIC-FAPI-04 was shown to be effective in distinguishing malignant lesions from benign lesions, suggesting its potential for the diagnosis of postoperative recurrence and metastasis of CRC. Moreover, ^99m^Tc-HYNIC-FAPI-04 uses the more widespread SPECT/CT for imaging, which is more conducive to clinical applications.

The major limitations of our study were the single-center study design and small study cohort. Larger studies are needed to validate the performance of dual-phase ^99m^Tc-HYNIC-FAPI-04 SPECT/CT.

## 5 Conclusions

Dual-phase ^99m^Tc-HYNIC-FAPI-04 SPECT/CT could be used to detect the metastatic lesions after surgery for CRC. It has a higher auxiliary role than single-phase imaging for the differential diagnosis of benign lesions and malignant lesions after surgery for CRC.

## Data Availability

The original contributions presented in the study are included in the article/Supplementary Material. Further inquiries can be directed to the corresponding authors.
